# α-Chitosan and β-Oligochitosan Mixtures-Based Formula for In Vitro Assessment of Melanocyte Cells Response

**DOI:** 10.3390/ijms25126768

**Published:** 2024-06-20

**Authors:** Verginica Schröder, Daniela Gherghel, Manuela Rossemary Apetroaei, Cristiana Luminița Gîjiu, Raluca Isopescu, Daniel Dinculescu, Miruna-Maria Apetroaei, Laura Elena Enache, Cosmin-Teodor Mihai, Ileana Rău, Gabriela Vochița

**Affiliations:** 1 Departament of Cellular and Molecular Biology, Faculty of Pharmacy, Ovidius University of Constanta, 6 Capt. Aviator Al. Șerbănescu Street, Campus C, 900470 Constanta, Romania; verginica.schroder@univ-ovidius.ro; 2 Institute of Biological Research Iasi, Branch of NIRDBS—National Institute of Research and Development of Biological Sciences Bucharest, 47 Lascar Catargi, 700107 Iasi, Romania; gabrielacapraru@yahoo.com; 3 Department of Marine Electric and Electronic Engineering, Faculty of Marine Engineering, Mircea cel Batran Naval Academy, 1 Fulgerului Street, 900218 Constanta, Romania; manuela.apetroaei@anmb.ro; 4 Faculty of Chemical Engineering and Biotechnologies, National University of Science and Technology POLITEHNICA Bucharest, 011061 Bucharest, Romania; luminita.gijiu@upb.ro (C.L.G.); raluca.isopescu@upb.ro (R.I.); laura.ena21@gmail.com (L.E.E.); ileana.rau@upb.ro (I.R.); 5 Faculty of Pharmacy, Carol Davila University of Medicine and Pharmacy, 6 Traian Vuia Street, 020956 Bucharest, Romania; miruna-maria.apetroaei@rez.umfcd.ro; 6 Praxis Medical Investigations, Moara de Vant St. 35, 700376 Iasi, Romania; mihai.cosmin.teo@gmail.com

**Keywords:** α-chitosan, β-oligochitosan, SK-MEL-28 melanocytes, YKL-40

## Abstract

Chitosan is a natural polymer with numerous biomedical applications. The cellular activity of chitosan has been studied in various types of cancer, including melanoma, and indicates that these molecules can open new perspectives on antiproliferative action and anticancer therapy. This study analyzes how different chitosan conformations, such as α-chitosan (CH) or β-oligochitosan (CO), with various degrees of deacetylation (DDA) and molar mass (MM), both in different concentrations and in CH–CO mixtures, influence the cellular processes of SK-MEL-28 melanocytes, to estimate the reactivity of these cells to the applied treatments. The in vitro evaluation was carried out, aiming at the cellular metabolism (MTT assay), cellular morphology, and chitinase-like glycoprotein YKL-40 expression. The in vitro effect of the CH–CO mixture application on melanocytes is obvious at low concentrations of α-chitosan/β-oligochitosan (1:2 ratio), with the cell’s response supporting the hypothesis that β-oligo-chitosan amplifies the effect. This oligochitosan mixture, favored by the β conformation and its small size, penetrates faster into the cells, being more reactive when interacting with some cellular components. Morphological effects expressed by the loss of cell adhesion and the depletion of YKL-40 synthesis are significant responses of melanocytes. β-oligochitosan (1.5 kDa) induces an extension of cytophysiological effects and limits the cell viability compared to α-chitosan (400–900 kDa). Statistical analysis using multivariate techniques showed differences between the CH samples and CH–CO mixtures.

## 1. Introduction

Melanoma is one of the cancers with increasing incidence worldwide [1]. The mechanisms that induce phenotypic plasticity of tumor cells [2,3], as well as the methods of therapy [1,4,5], are topics of great interest. Chemotherapy is less effective in this type of cancer [6], and resistance to therapy is a frequent issue. Therefore, alternative options are being sought to improve therapeutic outcomes and patient survival rates [5,7,8]. Using molecules of polymeric nature [1,4] or nanoparticles [1,5,9] targeting cancer cells or different intracellular components is a promising alternative.

Chitosan, obtained by deacetylation of chitin, is a potential drug delivery molecule used in targeted melanoma therapy [4,5,9]. Chitin (CT) and chitosan (CH) are among the most important biopolymers obtained from natural sources, including recycled waste, and are considered prospective molecules due to the diversity of applications [10,11]. Chitin is a homopolymer of N-acetyl-β-glucosamine (GlcNAc) and is the second most abundant polymer in nature. In vivo structural characteristics indicate that chitin is among the most resistant organic materials; this property is based on the molecular arrangement and the ability to combine with proteins or other components to form hybrid materials. In the evolved forms of eukaryotes and human cells, chitin structural units (chitobioses) are present at the level of glycoproteins or chitotriosidases, with a role in immune mechanisms in macrophages [12].

Chitosan is one of the biopolymers with applications in different fields, such as medicine, biotechnology, drug delivery, etc. [13,14,15,16]. Chitosan derives from various natural sources such as shellfish waste, molluscs, insect sloughs (exuviae), etc. The emerging modern research trends are identifying new sources, such as mollusc egg capsules [17,18], or developing methods to increase the efficiency of obtaining these materials [19,20]. Depending on the natural source and the processing methods of both the source and the chitin, chitosan acquires molecular versatility [21,22,23]. Thus, in acidic solutions, the amino groups of chitosan can be protonated, giving a polycationic behavior (at pH less than 6). At an increasing pH of around 6.6, the amino groups are unprotonated and form interpolymeric bonds that could induce polymer remodeling and interaction with proteins [24].

This behavior gives chitosan capabilities related to solubility in slightly acidic aqueous media and active bioadhesivity to build bonds with negatively charged substances, which are important features for understanding biological action. These particularities are related to the possibility of making forms of chitosan carriers with applications in the medical field, like drug delivery [25,26]. With breaking or swelling capabilities, the chitosan molecular network can be optimized for the coordinated release of active substances in the aqueous environment [27]. This biomaterial’s positive surface charge and biocompatibility allow it to effectively support cell growth, while its hydrophilic surface facilitates cell adhesion, proliferation, and differentiation [24,26,28].

Although these abilities make chitosan one of the polymers with the most studied applications, from a biological point of view, some phenomena limit the use of chitosan or low-deacetylating chitin-type polymers. The limitations of biological actions for these molecules relate to physicochemical characteristics such as solubility, viscosity, or molar mass of chitosan (CH) and chitin (CT).

The biological actions of chitosan (polymer or oligomer) can also be influenced by polymorphic forms. The conformation of the chains is modulated by temperature, ions, and pH so that cellular components (membranes, endosomes, lysosomes) can be targeted. Chitin polysaccharides can be of several types, α, β, and γ, differing in the arrangement of chains in the crystalline region [29].

Specific forms of chitosan or chitin from marine organisms are contingent upon the level of evolution [29]. Therefore, less-developed forms that have not yet been calcified could be significant sources of β-oligochitosan. On the other hand, evolutionary forms contain α-chitin, which, in its complex crystalline state, provides sea creatures with support and strength, found as mineralized shells [30]. Thus, based on the evolved forms of organisms, the different sources of chitin are represented by α-chitin, found mainly in the exoskeleton of crustaceans or insects; β-chitin, which has been identified in molluscs (squid) and algae (diatoms); while γ-chitin is found in fungi and yeasts [31,32,33]. After deacetylation, α- and β-chitosan can be used for developing biological applications. However, few studies can be found on the uses of β-chitosan due to its relatively limited availability, especially from squid species. Still, this compound is notable for its structural changes influencing solubility, swelling capacity, and biocompatibility [33].

Chitooligosaccharides (COS) have recently gained attention due to their synthesis through the degradation of chitin and chitosan. These include 2–20 units (β-(1-4)-N-acetyl-D-glucosamine and predominantly D-glucosamine residues) characterized by a low molar mass (less than 3900 Da). These compounds have been gaining much interest due to their increased solubility in water compared to chitin and chitosan [34,35].

The biological activity of these chitooligosaccharides is yet insufficiently established, as it depends on several parameters, such as polymerization degree, molar mass, deacetylation degree, acetylation fractions, and the origin of acetylation [19].

The β-(1-4)-glycosidic bonds between the N-acetylglucosamine and glucosamine units of chitin and chitooligosaccharides [23] are degraded by chitinase hydrolysis enzymes, which exist in different organisms, from viral forms, archaebacteria, bacteria, fungi, protists, and arthropods to plants and mammals. They are involved in many physiological processes, such as nutrition, parasitism, morphogenesis, and immunity.

Additionally, several inactive chitinases (chitinase-like lectins) are present in organisms. However, these lectins have no catalytic activity but a functional regulatory role. The best-known lectins belong to the 18 and 19 glycosyl hydrolase families of chitinases, and few belong to the 23 and 48 families, as identified in the last few years [36,37].

Hollak et al. detected the first human chitinase, which was found to have high chitinolytic activity in the serum of patients with Gaucher disease. Due to its ability to hydrolyze chitotriobase, it has been named chitotriosidase. Activated macrophages specifically express this protein and have a pH optimum of 6 [38].

Boot et al. discovered another chitinase with a catalytic capacity at pH 2, hence its name—acidic mammalian chitinase (AMCase). Genes for these enzymes have activity in the gastric epithelium, in lung macrophages, and in the lung epithelium on asthmatic inflammation [37,39].

These aspects suggest the existence of both chitooligosaccharide and chitin components in the extracellular and intracellular space, AMCase being active in lysosomes. The substrate probably reaches the cellular system in the form of chitooligosaccharides. Human chitinase-like proteins (CLPs) include YKL-40, YKL-39, and SI-CLP, molecules that are secreted by cancer cells, macrophages, neutrophils, synovial cells, and chondrocytes; of these, YKL-40 is the most investigated in cancer. This YKL-40 molecule is correlated with an inferior prognosis in breast, lung, prostate, liver, colon, and other cancers and is used as a predictive biomarker for tumorigenesis [40,41,42,43].

YKL-40 is also associated with an inflammatory effect in the intestine, for which a series of inhibitors, such as caffeine, theophylline, and pentoxifylline, may reduce the inflammatory process, thus showing promise in therapy. No selective small drug-like molecules that bind to the YKL-40 have been identified [37].

This study aims to evaluate how the polymorphic chitosan could modify the cell proliferation of a melanoma tumor culture (SK-MEL-28) by using α-and β-chitosan forms (polymer and oligomer) with different characteristics (deacetylation degree, DDA, molar mass, MM) and from different natural sources. For antiproliferative assay effects, in our study, α-chitosan from shrimp exoskeletons and β-oligochitosan from *R. venosa* egg capsule [17] mixtures were used. Our study hypothesized that the polymer mixture (α-chitosan–β-oligochitosan) induces various or synergistic interactions of biological activities. The intermolecular forces of β-chitosan are weaker than in α-chitosan, with these particularities making β-chitosan more soluble, reactive, and permeable [44]. Chitosan blending is a commonly employed technique for modifying polymers in terms of their functionality [29].

The present study also aims to identify the correlations between the chitosan forms and their influence on the expression of the chitinase-like protein YKL-40 on a melanoma cell line SK-MEL-28 (ATCC HTB-72). Studies have identified the affinity of YKL-40 for chitooligosaccharides [45], and experimental induction of these intracellular interactions could increase protein blockage. Taking into account the involvement of YKL-40 as a proinflammatory factor [46,47], its role in tumorigenesis [46,48,49], or in amplifying cancer progression (metastasis) [49,50,51], the alternative of inhibiting these proteins is a prospective direction.

## 2. Results

### 2.1. Physicochemical Characteristics of α-Chitosan and β-Chitosan

#### 2.1.1. Scanning Electron Microscopy of the Powders

**Figure 1:**
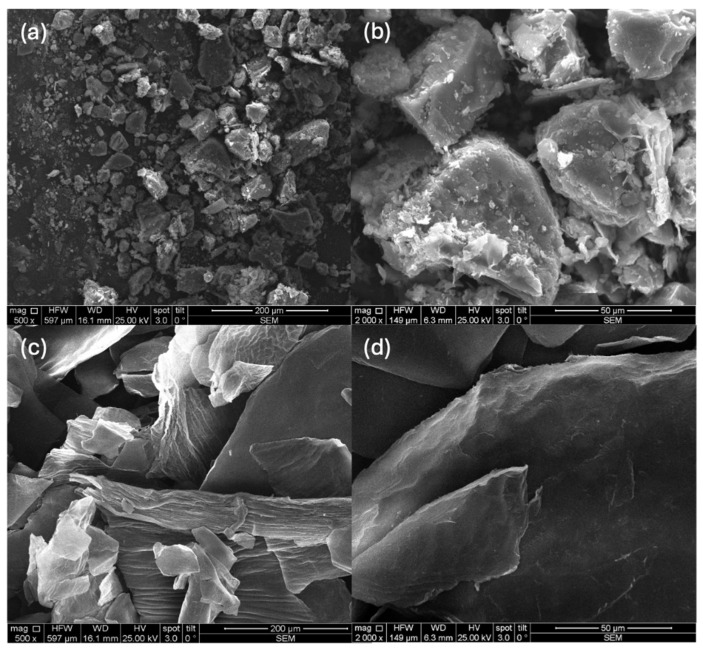
Details of powders analyzed by scanning electron microscopy (SEM); (**a**) β-oligochitosan magnification 500×; (**b**) β-oligochitosan magnification 2000×; (**c**) α-chitosan magnification 500×; (**d**) α-chitosan magnification 2000×.

Chitosan powders analyzed by scanning electron microscopy (FEI Company, Hillsboro, OR, USA) reveal particles with different appearances. Thus, β-oligochitosan is granular (Figure 1a,b), with rounded shapes and delicate overlapping layers. It has a semicrystalline appearance and rounded edges that can be distinguished in Figure 1b. The α-polymer particles (Figure 1c,d) have a compact, semicrystalline disposition but with very sharp edges. The data can be correlated with the results of our previous study, which indicated that X-ray diffraction (XRD) measurements of chitosan samples showed a reduction in the crystallinity index with DD increasing [52]. These details suggest the size and, respectively, the morphology of the particles generated by the bonds between the oligomeric β-chitosan matrix and the polymeric α-chitosan. Particle size and morphology are characteristics of particular interest in drug delivery and pharmacokinetics polymer applications [53,54].

Previous studies have shown that this chitosan has a β-type structure, semicrystalline with parallel polymer chains, characterized by a crystallinity index (33.53%) much lower than an α-type chitosan (40–80%) [55].

#### 2.1.2. Biopolymer Solutions

To identify the optimal formula for in vitro cell assays, the chitosan and oligochitosan powders extracted from marine wastes were characterized by DDA and MM. The data obtained are presented in Table 1.

For the first time, this study uses mixtures of α-chitosan and β-oligochitosan solubilized in 1% acetic acid solutions to obtain the optimal formula for cell growth with broad applications. Given their versatility and their chemical structure and different characteristics (DDA and MM), these blended biopolymers were developed to confer various applications, especially in the medical and pharmaceutical fields [56].

The chitosan mixtures used in this study were formed using the blending technique. This process of blending α-chitosan with β-oligochitosan involves the mix of two biopolymers that, despite their chemical similarity, exhibit different physical and chemical characteristics because of variations in their molar mass and degree of deacetylation. Regarding the chemical characteristics of the blending process, several interactions and modifications that could enhance or modify the characteristics of the new formulation have been considered.

The novel combination of α-and β-chitosan used in this study is based on the interactions between their molecular chains, favored by the following key parameters: molar mass, deacetylation degree, and structural arrangement. It is well known that the molar mass value influences the solubility of each type of chitosan. Thus, the combination based on mixing α-chitosan with β-chitosan provides an equilibrium between the strength and flexibility of the new formulations.

#### 2.1.3. Antioxidant Activity Assay

The antioxidant activity is reduced in the mixture compared to the nonblended samples, except for CHP1, where the mixture (MIX1) induces a minor change (from 13.28 ± 1.59% to 14.27 ± 1.81%) (Table 2). The polymer–oligomer interaction can explain the modification of the antioxidant activity; thus, a molecular remodeling occurs via the -OH or -NH_2_ groups, initially unavailable for the anti-free radical’s effect. The results indicate lower antioxidant activity of the tested samples.

### 2.2. Biological Effects

#### 2.2.1. Evaluation of the Effects on Cell Viability by the MTT Method

**Figure 2:**
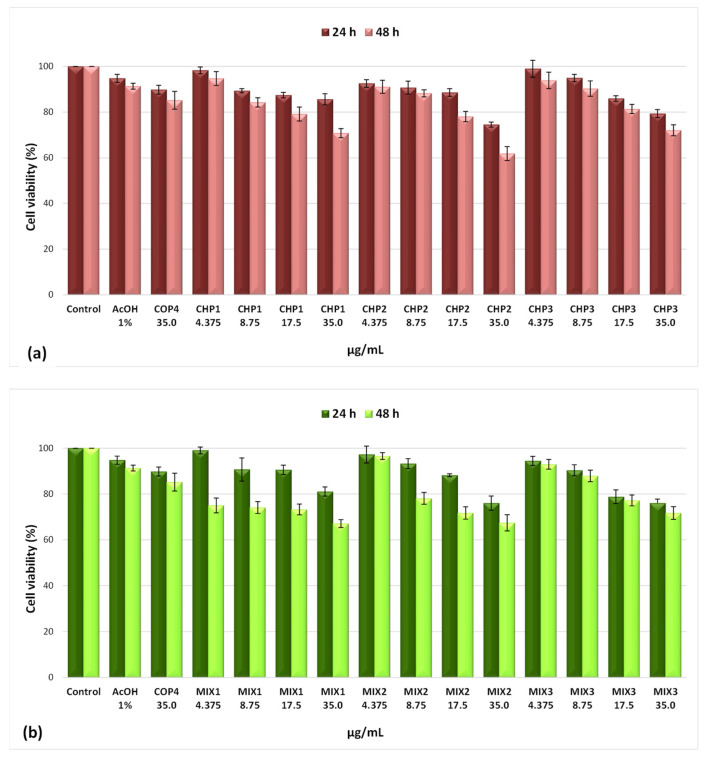
SK-MEL-28 cell viability after 24 h and 48 h treatment with different chitosan polymers—CHP1, CHP2, CHP3—(**a**) and mixtures—MIX1, MIX2, MIX3—(**b**); as well as acetic acid (AcOH) and oligomer (COP4) (mean values and SD).

The MTT viability assay revealed a decrease in the viability of SK-MEL-28 melanoma cells with the increase in the tested doses and in the treatment duration. Thus, at the minimum tested concentration (4.375 µg/mL), cell viability after 24 h of treatment varied from 99.08% in the presence of the oligomer (MIX1) to 92.57% when the CHP2 polymer was added. The viability of SK-MEL-28 cells at 48 h, under the action of the minimum tested dose, ranged between 96.60% (MIX2) and 75.04% (MIX3), proving an increase in the cytotoxic effect. A more pronounced interference with cell viability was observed as the dose increased at the maximum concentration (35 µg/mL) with values ranging from 85.62% (CHP1) to 74.58% (CHP2) after 24 h of treatment. Significant decreases in cell viability were observed after 48 h of contact of SK-MEL-28 cells with the studied solutions, with values of 72.03% (CHP3) and 61.85% (CHP2). All these aspects are illustrated in Figure 2a,b.

Pursuant to the cell viability test, it was found that the formulations with oligochitosan showed a significant cytotoxic effect after 48 h of treatment and at the maximum dose. In the case of the MIX2 combination, a slightly higher viability value was recorded (67.46%), compared to 61.85% determined in the case of the CHP2 polymer. The most bioactive sample was MIX1, with cell viability of 67.11%, compared to the 70.83% value found in the case of polymer CHP1. The solvent used, 1% acetic acid, did not have a negative effect on cell viability (91.36%), thus proving its nontoxic nature. The degree of cytotoxicity of the oligomer (COP4) was insignificant, registering a cell viability value of 85.20% compared to the untreated control (100%).

The IC_50_ values indicate slight changes in the effect at 24 h of exposure to the CHP1 compound (88.74 µg/mL) compared to CHP2 (78.45 µg/mL) and CHP3 (73.98 µg/mL). Similar values were obtained in the case of mixtures, varying between 73.40 µg/mL (MIX2) and 75.01 µg/mL (MIX1 and MIX3). After 48 h of exposure, differences between the samples are noticeable without showing a clear relationship with sample type (Table 3).

#### 2.2.2. The Cell Morphology Test

Evaluation of the interaction of different chitosan formulas with the morphology of SK-MEL-28 cells revealed that the main changes at the cytomorphological level consisted of the reduction in the ability to adhere and to form a cell monolayer.

**Figure 3:**
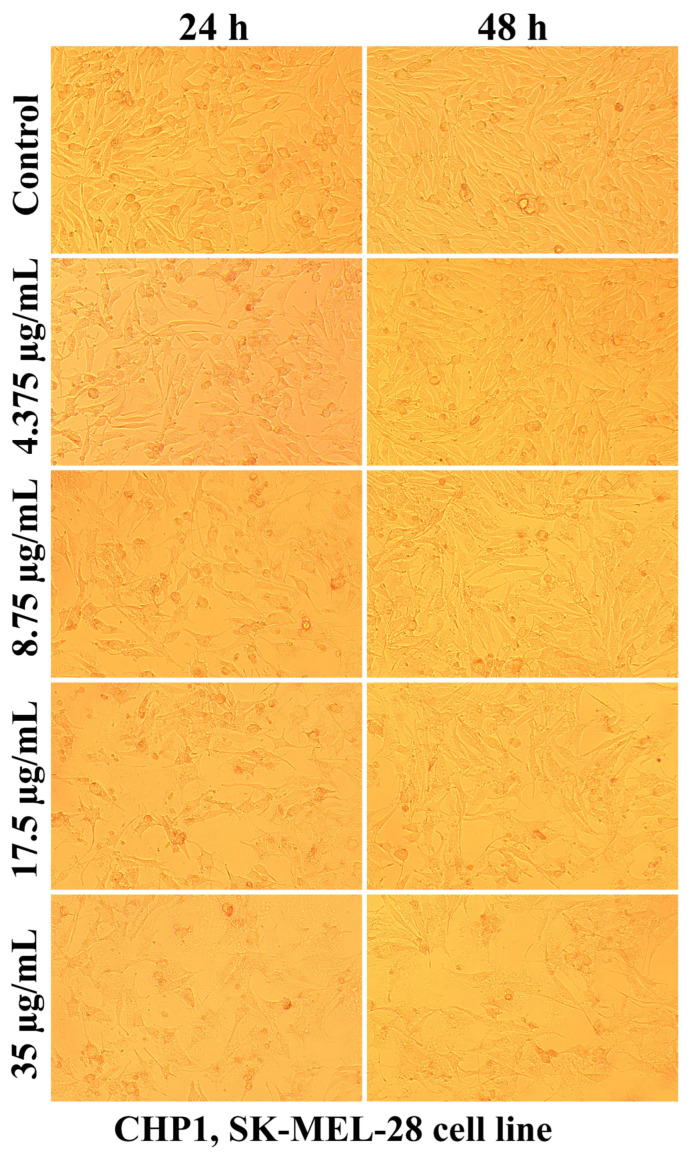
Morphological aspects of SK-MEL-28 human melanoma cells after CHP1 treatment for 24 and 48 h.

**Figure 4:**
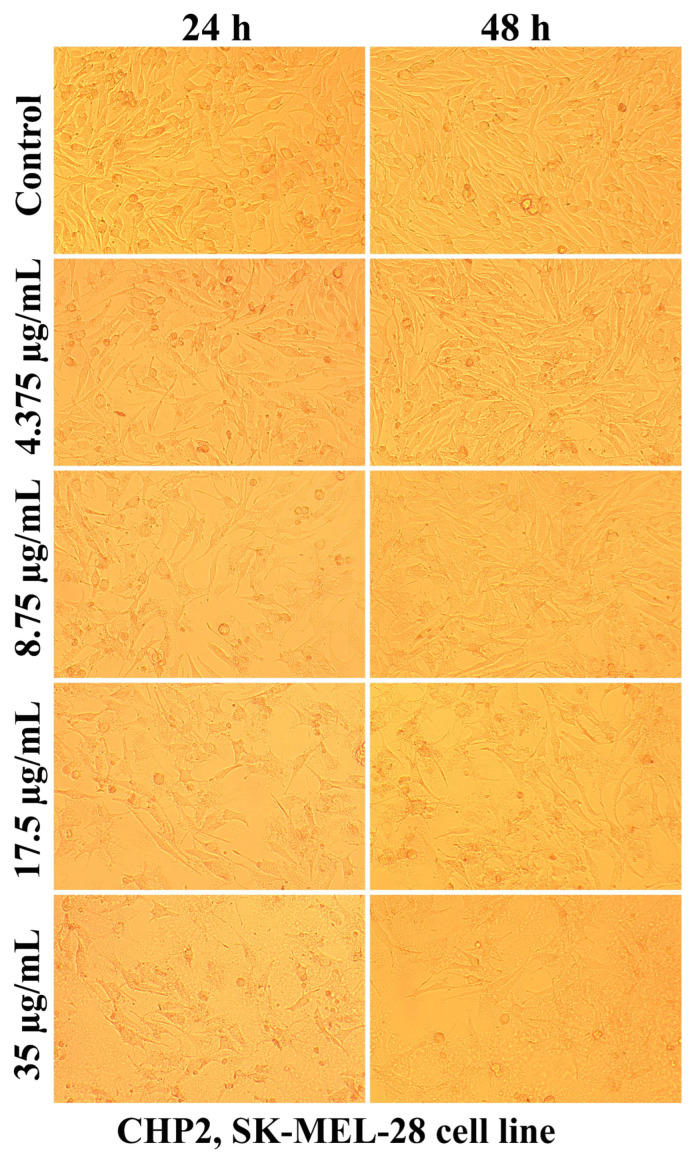
Morphological aspects of SK-MEL-28 human melanoma cells after CHP2 treatment for 24 and 48 h.

**Figure 5:**
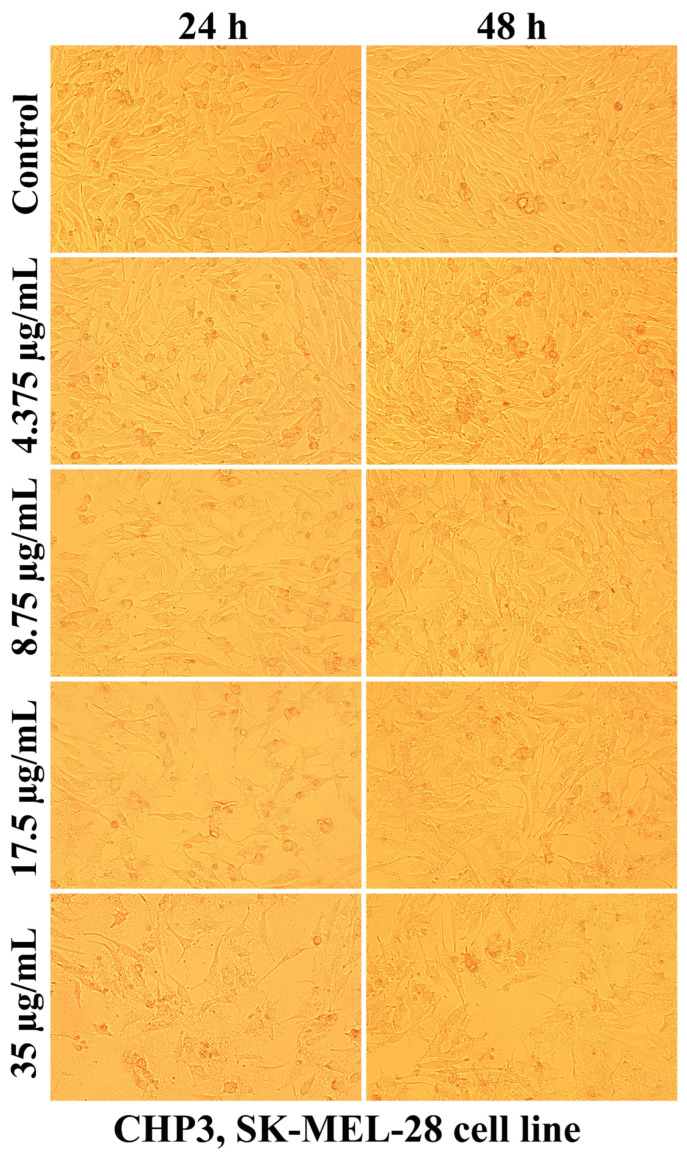
Morphological aspects of SK-MEL-28 human melanoma cells after CHP3 treatment for 24 and 48 h.

**Figure 6:**
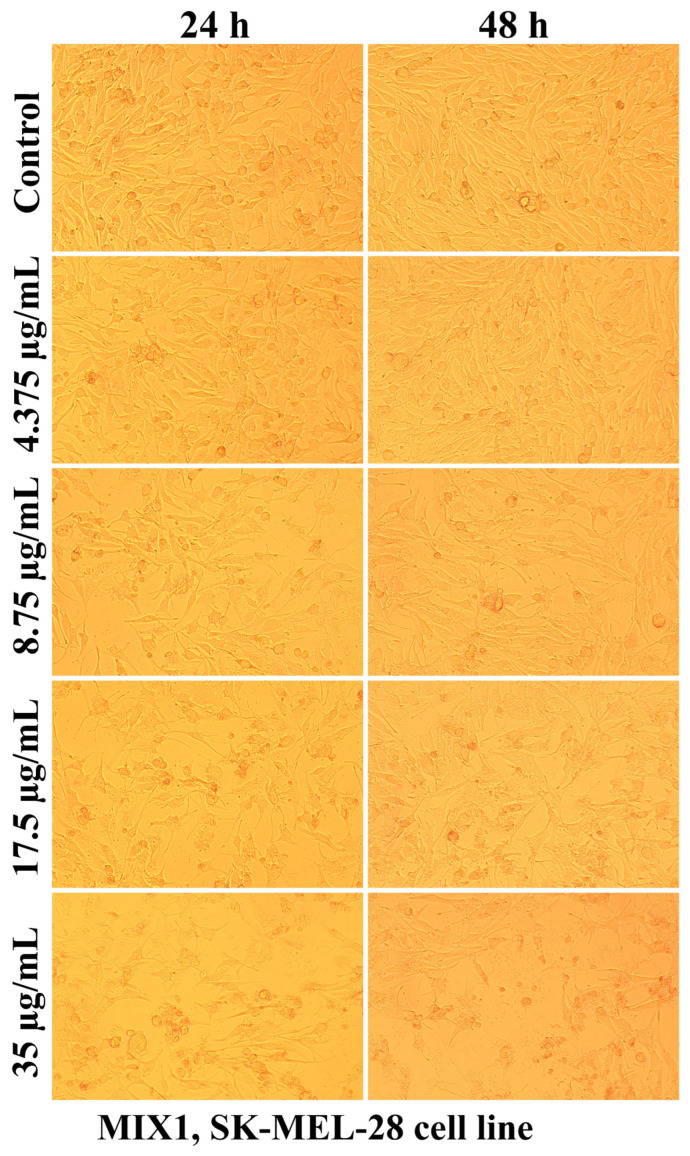
Morphological aspects of SK-MEL-28 human melanoma cells after treatment with MIX1 for 24 and 48 h.

**Figure 7:**
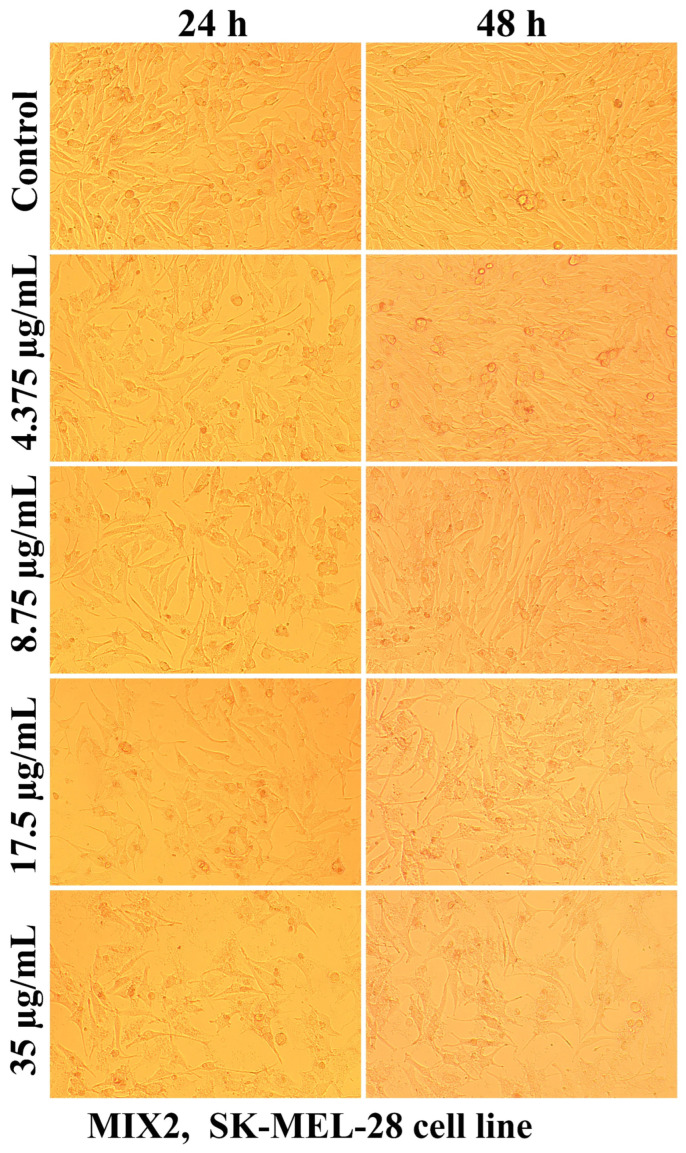
Morphological aspects of SK-MEL-28 human melanoma cells after treatment with MIX2 for 24 and 48 h.

**Figure 8:**
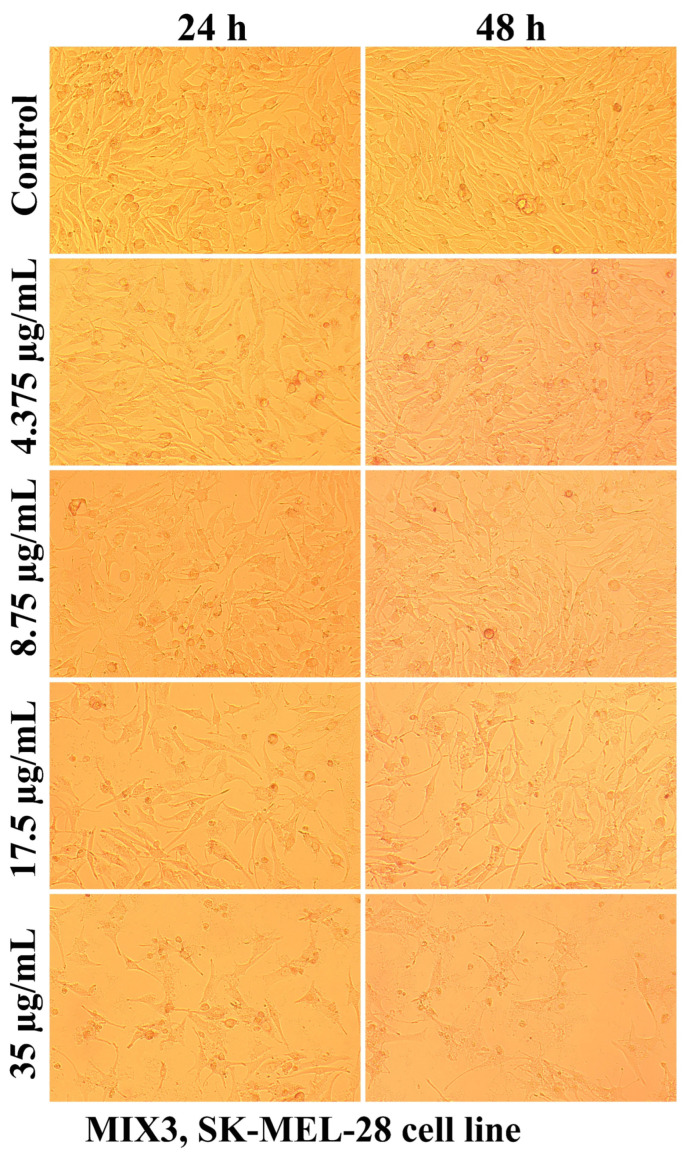
Morphological aspects of SK-MEL-28 human melanoma cells after treatment with MIX3 for 24 and 48 h.

The loss of the intercellular connections that ensured the unity of the monolayer leading to the reduction in the number of confluent cells and the appearance of isolated cells, more or less spherical floating in the culture medium, was much more intense at the maximum tested concentration (35 µg/mL) and after 48 h of treatment (Figure 3, Figure 4, Figure 5, Figure 6, Figure 7 and Figure 8).

#### 2.2.3. Determination of YKL-40 Protein Expression

**Figure 9:**
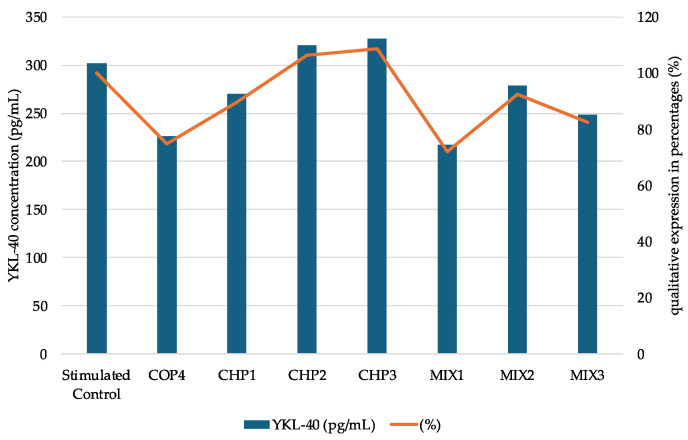
YKL-40 levels (pg/mL) samples interpolated from the standard curve and percentage expression of samples compared with control; SK-MEL-28 human melanoma cells LPS stimulated and α-chitosan (CHP1, CHP2, CHP3) and α-chitosan–β-oligochitosan (1:2) (MIX1, MIX2, MIX3) samples treatment (mean values); qualitative expression percentages among the samples and the control (LPS stimulated).

YKL-40 is a glycoprotein synthesized by cells in response to microenvironmental factors, which induces the modeling of cellular activity [16]. In the present study, the evaluation of YKL-40 activity led to the recording of some quantitative variations in the level of this protein. After lipopolysaccharide (LPS) stimulation, SK-MEL-28 cells increased glycoprotein synthesis by 9.27 pg/mL, representing an increase of 3.17%, compared to the unstimulated cells. According to the results after the application of the mixtures (Figure 9), the level of YKL-40 decreases in the case of the tested solutions by 7% for MIX2, 18% for MIX1, and 28% when administering the MIX3 mixture compared to the control sample. The inhibition effects of YKL-40 synthesis are related to the presence of chitosan oligomers. In the samples without oligochitosan, increases in the level of YKL-40 by 9% (CHP2, LPS stimulated) and 12% (CHP3, LPS stimulated), respectively, were found. No changes were noted that correlate with the particularities of the polymer, which denotes similar mechanisms of molecular stimulation in the cell.

YKL-40 expression is identified at the cytoplasmic level, or, in the case of tumors, in the extracellular matrix, which denotes its exocytosis while maintaining the negative cell membrane [17]. The study on several types of tumor cells highlighted the in vitro low expression of this protein, which is also pointed out in our studies, the evaluations being of the order of pg/mL.

### 2.3. Statistical Analysis of Experimental Data

The results obtained using chitosan and oligochitosan mixtures were statistically analyzed using principal component analysis (PCA) and ANOVA.

A global view aiming to emphasize the differences between the six different experimental conditions according to the use of three chitosan samples (CHP1, CHP2, and CHP3) and their mixtures with oligochitosan, denoted by MIX1, MIX2, and MIX3, respectively, was carried out using PCA. These pure chitosans and blended biopolymers were characterized by six variables linked to their main characteristics (DDA and MM) and by the effects in biological tests: the enhancement of glycoprotein YKL-40 and the IC_50_ indexes for a contact time of 24 h (IC_50_-24) and 48 h (IC_50_-48) during the treatment of SK-MEL-28 cells as well as the antioxidant capacity (DPPH). The DDA value of the mixtures was calculated (Equation (1)) considering the mass ratios chitosan/oligochitosan (1/2) used and the DDAs of chitosan and oligochitosan, taking into consideration that the basic chitin–chitosan dimer is the same in all structures.

(1) $$DDA_{\mathrm{mix}}=\left(DDA_{\mathrm{chitosan}}+2\cdot DDA_{\mathrm{oligochitosan}}\right)/3$$

The mean molar mass of the mixtures was calculated using mol base, considering the molar mass of chitosan and oligo-chitosan and the mass ratio in the mix, which is 1/2 (Equation (2)).

(2) $$MM_{\mathrm{mix}}=3/\left(1/MM_{\mathrm{chitosan}}+2/MM_{\mathrm{oligochitosan}}\right)$$

**Figure 10:**
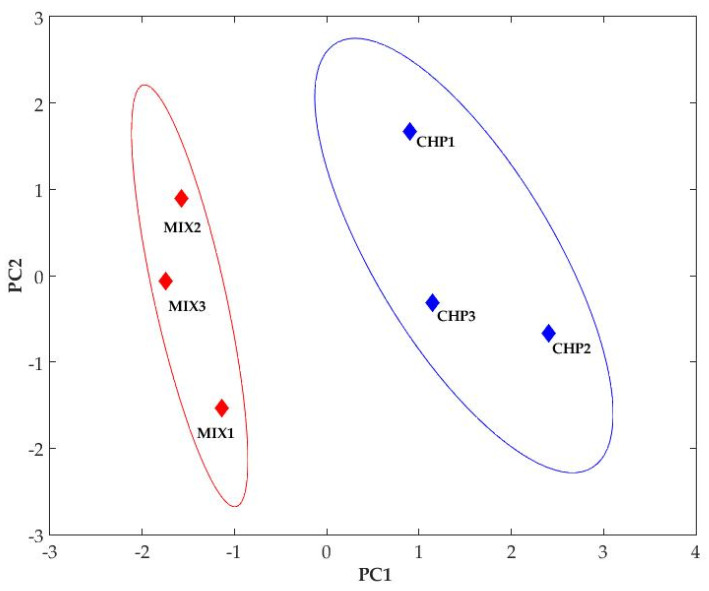
Samples representation in the first two PCA components.

According to PCA, the six variables implied were grouped in principal components (PCs), which encompasses the variability in the dataset in decreasing order and also shows a possible grouping of the samples. The first three PCs reflect about 90% of the variability (PC1—49%, PC2—22%, PC3—21%), and the representation of the samples in PC1–PC2 coordinates (Figure 10) shows that the chitosan samples are clearly separated from the mixture samples.

Analyzing the variable loadings on PC1 and PC2 as expected, the highest loading in PC1 corresponds to the MM, which drastically varied between the samples, followed by the loading of DPPH (91% from the loading of MM) and DDA and YKL40, about 80% from the loading of MM. IC_50_-24 h and IC_50_-48 h have high loadings in PC2 and PC3, respectively. This analysis demonstrates that the sample differentiation is made by modifying MM and DDA due to mixing and, to a considerable extent, due to the different effects in the biological system (YKL-40 synthesis and antioxidant capacity).

**Figure 11:**
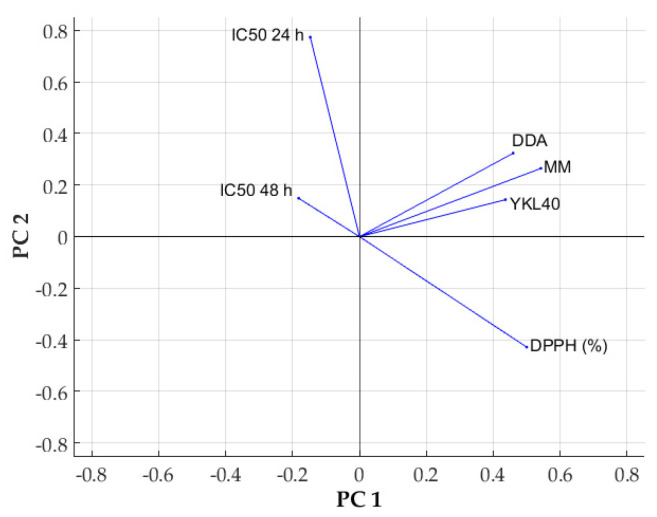
Variable loadings on the first two components.

Figure 11 depicts the relative importance of variable loadings and proves the positive correlation between MM and YKL-40, MM and DPPH, with DDA and DPPH standing for the decrease in protein YKL-40 when the molar mass is lower and increase in antioxidant capacity at higher deacetylation degrees. Therefore, the mixture chitosan–oligochitosan can improve both the antioxidant capacity and the DDA of the mixture, which is enhanced by the presence of chitosan. At the same time, the molar mass is mainly influenced by the oligomer, which leads to an important decrease in the YKL-40 generation.

The experimental viability of SK-MEL-28 cells treated with various solutions containing chitosan and chitosan–oligochitosan mixture was further analyzed using the factor analysis technique. The factors examined are the biopolymer type, the solution concentration (4.375, 8.75, 17.5, and 35 µg/mL), and contact duration (24 and 48 h). The three-way ANOVA performed in the frame of Matlab2023 (Table 4) shows *p*-values for all three factors lower than 0.05, which stands for their significance in the variation of the cells’ viability.

The interaction between time and polymer type is also important (*p* = 0.0096), which means that not all polymers have the same influence when the duration of the treatment varies (also reflected in Figure 2). Consequently, a two-way ANOVA was performed considering the influence of polymer type and concentration separately for the two process durations (24 and 48 h). For the samples analyzed after 24 h, the two-way ANOVA test (Table 5) shows the significance of both concentration and polymer type, and there is no interaction between the two factors.

**Figure 12:**
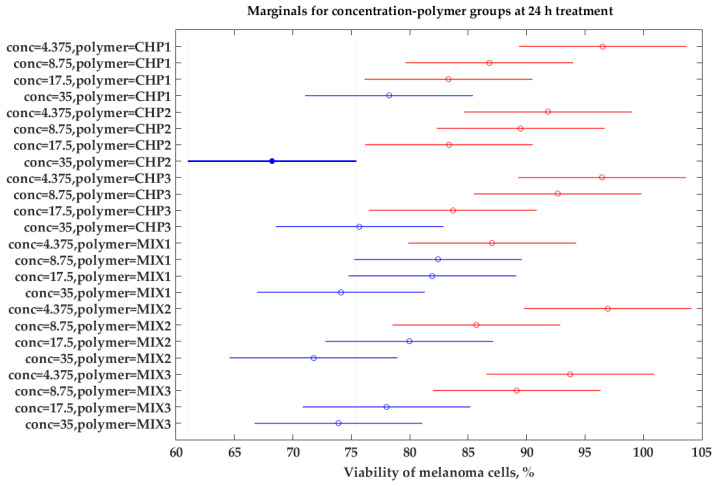
Marginals for sample reflecting melanoma cells’ viability for different oligopolymers and solution concentrations.

The post hoc Tukey test allows us to highlight more critical differences between the means of all possible pairs using a studentized range distribution. Figure 12 shows a graphical representation of the differentiations between groups and shows that the lowest SK-MEL-28 cell viability is registered for high concentration (35 µg/mL) using polymer CHP2, the thicker blue line. The blue lines represent groups with similar results and correspond to other chitosan polymers at a high concentration (35 µg/mL) and the chitosan–oligochitosan mixtures at lower concentrations (17.5 and 8.75 µg/mL). The red lines represent all other samples that are significantly different. This may support the assumption that for a contact time of 24 h, the mixtures of chitosan and oligochitosan may have better results.

For the experimental data obtained at 48 h contact time, two-way ANOVA proved significant effects for both main factors and interaction, imposing a further analysis focused on evaluating the polymer type effect at each concentration.

This study proved that at low concentrations (4.75 and 8.45 µg/mL), the biopolymer type has an important influence on the measured viability. In contrast, at higher concentrations (17.5 and 35 µg/mL), the changes in the biopolymer used made no significant differences in the viability of melanoma cells (Table 6).

This result may lead to the conclusion that all biopolymers can have comparable effects at high solution concentrations and prolonged contact time. In contrast, at shorter contact time and/or low solution concentrations, the nature of the biopolymer has a significant effect, with the chitosan–oligochitosan mixture being more effective in treating melanoma cells.

**Figure 13:**
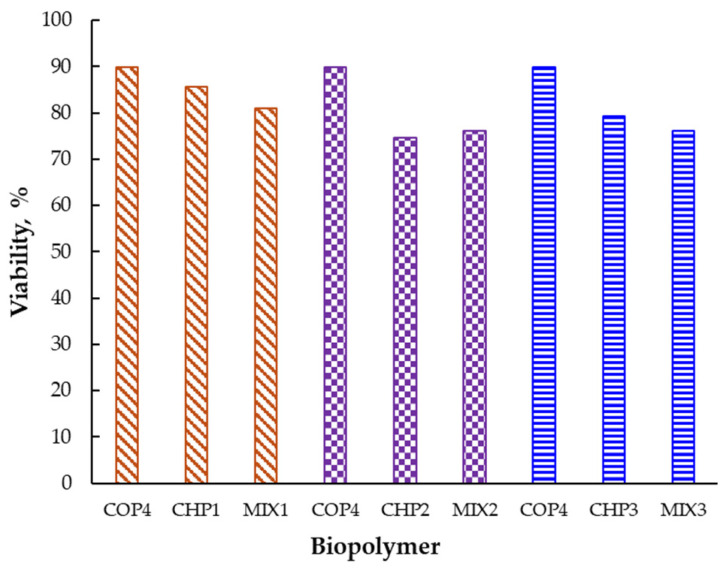
Mean viability of SK-MEL-28 cells after 24 h of treatment with 35 µg/mL solutions of oligochitosan (COP4), chitosan (CHP1, CHP2, CHP3), and the corresponding composite (MIX1, MIX2, MIX3).

Analyzing the mean viability of SK-MEL-28 cells using a concentrated solution of 35 µg/mL of chitosan, a chitosan–oligochitosan mixture, and oligochitosan, it can be easily noticed that the mixture gives better results than both chitosan and oligochitosan, which stands for the synergetic effect of the oligomer–biopolymer compounds in defining the biological properties (Figure 13).

As Figure 13 shows, oligochitosan alone is the least efficient, but combining it with chitosan can enhance its potency.

## 3. Discussion

Using chitosan with different characteristics modeled by the obtaining methods (DDA and MM) emphasizes that the molecules behave differently due to the presence of amino and acetyl groups. The degree of deacetylation influences the density of amino groups available for ion-bonding interactions, either with other chitosan molecules or within chitosan chains. Using chitosan with different deacetylation degrees (DDA) could improve the newly formed material’s solubility, charge density, and bioactivity. The structural arrangement of the new formulation, through changes brought about by the presence of β-chitosan, such as differences in chain flexibility or the presence of branching, could explain how these polymer molecules interact with chitosan matrices or gels. In this way, the different interactions and biological effects purchased with pure chitosan can be explained.

This is due to reactive functional groups in the glycosidic ring structure: the primary hydroxyl group at C^3^, the secondary hydroxyl group at C^6^, and the reactive amino group at C^2^, which are involved in inter- and intramolecular bonding. Data in the literature show that these reactive functional groups provide the basis for enhancing chitosan functionalization by modifying the chemical structure of chitosan through alkylation, acylation, esterification, oxidation reactions, etc. The resulting functionalized chitosan-type products exhibit higher selectivity than their nonfunctionalized counterparts and, thus, can be used in a broader range of applications [29,57].

Chitosan is studied for its various biological properties: antitumor, antimicrobial, antioxidant, and anti-inflammatory. The polymer’s physicochemical characteristics, such as molar mass, DDA, concentration, or solubility, are the main intrinsic factors influencing effects in biological systems. Intracellular oxidative stress causes necrosis and cell degradation, but the cell is equally adapted to maintain a redox balance. Understanding how these intracellular relationships can be manipulated may provide an advantage in preventing and treating diseases [58].

The antioxidant activity of chitosan is related to its ability to bind free radicals, an activity that depends on active hydroxyl and amino groups in their polymer [59,60]. Various methods used to analyze scavenger capacity show that the level of antioxidant activity depends on the concentration level of chitosan or oligochitosan as well as molecular weight [10,59,60].

The maximum concentration tested of chitosan (35 μg/mL) showed that scavenging activity is below 20%. Other studies looking at chitosan, chitosan oligomers, or its derivatives at concentrations below 1 mg/mL have demonstrated similar results [59,61,62]. Chitosan oligomers can have a scavenging effect of only 7% at MM of about 15 kDa [59].

Although the level of antioxidant activity is low, for our study, it represents an advantageous response in the sense that at the tested concentrations and in the combinations used in this experiment, the antioxidant activity indicated in vitro preserves the conditions of cell availability, favoring the desired cytotoxic and antitumor effects.

Chitosan of different origins and characteristics, with MM ranging from 1.5 KDa (COP4) to 2.25 KDa in mixtures (MIX 1, MIX2, MIX3) to 819.99 KDa (CHP1), 804.33 KDa (CHP2), and 475.43 KDa (CHP3), induces similar mechanisms at the same concentration, which changes the perspective of understanding the level of versatility of chitosan. Numerous studies are searching for solutions to limit melanoma cell proliferation by targeting some matrix components or intracellular structures. Also, combined therapies such as chemotherapy, radiotherapy, or immunotherapy are explored [7]. Our studies have proven a decrease in cell viability after 24 and 48 h for all the experimental variants compared to the untreated control. Mixtures with oligochitosan favor changes that attract attention through significant differences regarding cell viability. There are several possibilities by which these changes are accomplished: either the oligochitosan interacts with other membrane or intracytoplasmic components, or there are molecular changes of the oligomer by organizing into micelles, along with the change in intracellular pH [12,63].

The mechanisms of action of chitosan and oligochitosan or mixtures occur in successive stages. In the first stage, we appreciate that interaction with cancer cell membranes occurs. These interactions induce changes in the morphology and adhesion of cells (Figure 3, Figure 4, Figure 5, Figure 6, Figure 7 and Figure 8), observed in our experimental conditions.

Chitosan is a polymer with amino groups, which are positively charged and will react with the negative charges of membrane surfaces. These interactions may explain the differences in the response of different cell lines in contact with chitosan molecules or oligochitosan [64].

The mentioned electrostatic interactions can be correlated with the higher negative charges on the membranes of cancer cells compared to normal cells, hence the variability of chitosan cytotoxicity on different cell lines [65]. A study on the influence of chitosan on melanoma revealed a decrease in the proliferation of primary melanoma cells, the SK-MEL-28 line, as well as cells derived from metastatic lymph nodes, the RPMI-7951 line, by activating the proapoptotic pathways. In the same study, another cell line, A-375, also a primary melanoma, skin-derived cells, does not show the same proliferative changes as SK-MEL-28, without the exposure time being correlated with the change in the proliferation of these cells [66].

The effects of these chitosan molecules are correlated with the regulation of proliferative processes, more precisely intervening in cell signaling through the phosphatidylinositol 3-kinase (PI3K)-AKT pathway, one of the most important signaling pathways associated with tumorigenesis and cancer treatment. According to other studies in this direction, there are controversial opinions. Amirani et al. highlighted the anticancer role of chitosan and oligochitosan by affecting PI3K-AKT [67].

Another study points out the intracellular manifestation of chitosan with the role of stimulating the PI3K/AKT1/mTOR pathway. The amplified expression of proteins that increase cell proliferation in the presence of chitosan nanoparticles (DDA = 75–80%) in liver cancer cells, CCL 13 and HepG2, can conclude that the use of the polymer with 75–85% deacetylation and low molar mass presents a risk regarding the use of chitosan in antitumor applications on these cells [14]. Another study reveals the anticancer activity of oligochitosan by inducing some electrical changes [68].

The observations from our study denote the correlation between the particularities of chitosan molecules and the amplification of cytotoxic effects. Thus, chitosan with high DDA (CHP1) has an impact of inhibiting cell viability. Still, the decrease in MM leads to the acceleration and maintenance of the effect (CHP3), a phenomenon explained by the increase in the rate of penetration and possible accumulation at the endosomal level. Cancer cells use different adaptations in the malignancy process, including inhibiting some antiapoptotic or antioxidant pathways induced by various chemical substances used in therapy. Activation of these cellular signaling pathways leads to drug resistance and stimulation of proliferation. Chitosan acts as a scavenger through the hydrogen ion of NH_3_ from the C^2^ position. The mechanism of the antioxidant activity induced by chitosan is influenced by the physical–chemical characteristics (DDA or MM). Still, these effects are also related to the concentration of the polymer molecules [69,70].

Many studies have tried to elucidate the mechanisms of chitosan’s action. However, it has been concluded that this polymer and its derivatives produce different responses in different cell types [65]. The quantified effects (IC_50_) and concentrations used are also very varied. Thus, Zou et al. [64] report, in their studies, that the IC_50_ value of oligochitosan was observed for different cell lines: IC_50_ = 1329.9  ±  93.4 µg/mL for HCT-116, IC_50_ = 48.6  ±  7.0 µg/mL MCF-7, respectively [65], IC_50_ = 800 ± 131.45 µg/mL on Ca9-22, but not HaCaT cells [71]. Srinivasan et al. reported the lowest concentration of chitosan (10 μg/mL) used in vitro ovarian cell testing (ovarian cancer cell line—PA-1) with an antitumor effect of 100% [72]. Another study showed that the highest concentration of oligochitosan tested was 40 mg/mL [73] on HeLA cells, with morphological effects. As a result, chitosan concentration is another variable parameter that is not very well understood.

The decrease in SK-MEL-28 cell viability after applying different chitosan-based polymers was correlated with cytomorphological changes, such as reduced cell adhesion, the appearance of floating cells in the culture medium, thinning of pseudopods, and widening intercellular spaces.

Cultivation of some colon cancer cell lines and hepatocellular carcinoma cells on chitosan and hyaluronan grafted chitosan membranes determined alterations of the normal morphology (monolayer formation) materialized by the formation of spheroids or colonies as a result of 72 h treatment with chitosan-based membranes [74].

The YKL-40 (Chitinase-3-like protein 1) molecule, under experimental conditions, initiates cell proliferation through the ERK1/2-MAPK pathway and supports angiogenesis through the interaction of syndecan-1 (SDc-1/αVβ3) and with receptors (IL-13Rα2) at the level of endothelial cells. It also stimulates metastasis by activating proinflammatory cytokine-type factors (TNF-alpha, interleukin-1beta, interleukin-6, interferon-gamma) and proinvasive factors (MMP9, CCL2, CXCL2) [75,76]. YKL-40 is a lectin that binds carbohydrates with a preference for chitin, heparin, and hyaluronic acid. YKL-40 is synthesized and secreted by many cells (macrophages, neutrophils, synoviocytes, chondrocytes, fibroblast-like cells, smooth muscle cells, and tumor cells). It plays an important role in the cellular response to environmental changes (tissue damage, inflammation, tissue repair). YKL-40 has been shown to be overexpressed in a variety of human and animal cancer models with roles in cancer cell growth, proliferation, invasion and metastasis, angiogenesis, activation of tumor-associated macrophages, and Th2 polarization of CD4+ T cells [77]. That is why YKL-40-based targeted therapy is increasingly being approached in cancer pathologies [50,51].

The in vitro stimulation of YKL-40 synthesis in samples with LPS and samples with α-chitosan and LPS indicates that SK-MEL-28 melanocytes react to the introduction of β-oligochitosan in the mixture. According to other studies, these molecules may show an affinity for YKL-40, which blocks the release into the extracellular space. It should also be mentioned that chitosan–oligochitosan concentrations that induced a significant decrease in cell viability (IC_50_) were used for this test, which means that cytotoxic effects can be considered. The SK-MEL-28 melanoma line is characterized by a low expression level of YKL-40 [44]. Regarding skin cancers, the SK-MEL-28 line is among those with a low value of nTPM values (representing the number of RNA transcripts per million) of 0.6 for the gene responsible for transcribing the YKL-40 protein [78].

Through these observations and statistical analysis of the results, the action of chitosan can be anticipated and correlated with doses and combinations that favor the desired processes. Statistical analysis showed significant changes in melanoma cells at low concentrations of chitosan in the first 24 h (Table 6), while high concentrations did not correlate with chitosan types.

It is possible that chitosan might have a sensibilization activity on the cells and could, thus, through controlled molecular characteristics, become a therapeutic agent in combination therapies [79] targeting cellular components.

A number of experiments highlight the versatility of chitosan and its use in various anticancer applications, either as codelivery of oxaliplatin and rapamycin for synergistic chemotherapy [80] or by triggering other mechanisms, such as inhibition of the permeation-glycoprotein (P-gp), a protein with an important role in the management of cancer [81].

Our study showed that mixing (alpha and beta forms) of chitosan can induce changes in cellular responses (metabolic, morphologic, and protein synthesis). Further to cytotoxicity, the presence of oligomer leads to the synthesis of YKL-40 (Figure 9), a protein involved in various cellular processes and angiogenesis. Inhibition of angiogenesis is the focus of modern antitumor studies [82]. Our study, thus, allows the observation of a correlated process between chitosan and melanoma cell response, while the mathematical modeling (Figure 10, Figure 11, Figure 12 and Figure 13, Table 6) is useful to classify quantified processes and differentiate them based on statistical significance and the dynamics of their appearance in the cells.

## 4. Materials and Methods

### 4.1. Materials

According to Dinculescu et al. [83], chitosan powders (CHP1, CHP2, CHP3) were obtained by optimizing shrimp waste through a recovery process. The oligochitosan powder (COP4) was chemically extracted from *Rapana venosa* capsule waste, aligning with the previous study’s methodology [30].

The other chemical reagents employed were as follows: HCl (Chemical Company S.A., Iasi, Romania), NaOH pellets (ChimReactiv SRL, Bucharest, Romania), ethyl alcohol (Sigma Aldrich, Taufkirchen, Germany), acetone (p.a.) (Sigma Aldrich, Taufkirchen, Germany), acetic acid solution (Sigma Aldrich, Taufkirchen, Germany), methanol (Merck KGaA, Darmstadt, Germany), dimethyl sulfoxide (DMSO) (Merck KGaA, Darmstadt, Germany), trypsin/EDTA (Biowest, Nuaillé, France), 3-(4,5-dimethylthiazol-2-yl)-2,5-diphenyltetrazolium bromide (MTT) (Merck KGaA, Darmstadt, Germany), 2,2-diphenyl-1-picryl-hydrazyl-hydrate (DPPH) (Aldrich Chemistry, MM:394.32, Steinheim, Germany), melanoma cell line SK-MEL-28 (ATCC HTB-72), Eagle’s Minimum Essential Medium (PAN-Biotech GmbH, Aidenbach, Germany), fetal bovine serum (SFB, Euroclone S.p.A., Milan, Italy), antibiotic solution (mixture of penicillin 100 μg/mL and streptomycin 100 IU/mL—Capricorn Scientific GmbH, Ebsdorfergrund, Germany), lipopolysaccharide from *Escherichia coli* O55:B5 (MedChemExpress, Monmouth Junction, NJ, USA), CHI3L1 ELISA Kit (antibodies-online GmbH, Aachen, Germany).

### 4.2. Synthesis and Characterization of Chitosan Samples

The extraction procedure of chitosan powders [84,85] involved a demineralization step, using 4% HCl solution in the ratio of shrimp waste powder to HCl solution of 1:13 (*m*/*v*) at room temperature for 50 min. The deproteinization step involved a 5% NaOH solution in the mixture ratio of demineralized powder to NaOH solution of 1:16 (*m*/*v*), at 65 °C, under continuous stirring for two hours. Prior to the deacetylation stage, the obtained chitin powder was subjected to a decolorization process using a mixture of ethyl alcohol and acetone in a volumetric ratio of 1:1 (*v*/*v*) at room temperature. The deacetylation procedure of the obtained chitin samples was carried out with 45% NaOH solution for 120 min under continuous medium agitation and in a solid-to-liquid ratio of 1:18 for CHP1 and CHP2 and in a solid-to-liquid ratio of 1:9.5 for CHP3. Following each stage of the above-specified procedure, the material was rinsed with distilled water until achieving a neutral pH. Subsequently, the material was dried to a constant weight. By using different solid–liquid ratios (m_chitin_:vol_NaOH sol_) under the same temperature conditions (95 °C), contact time (120 min), and the same NaOH concentration, it was desired to obtain chitosan samples with high values for DD, but with different values for molar mass. Thus, at ratios of m_chitin_:vol_NaOH sol_ =1:9.5, in the case of the CHP3 sample, the measured molar mass was almost half that of CHP1 and CHP2, which were obtained at ratios of 1:18 (solid–liquid).

According to our previous study [30], the oligochitosan powder (COP4) was obtained by chemical extraction of *R. venosa* capsule waste using a 6.5% NaOH solution in a capsule mass: NaOH solution ratio of 1:40 (*m*/*v*) at 90 °C, under medium agitation, for 120 min. The obtained alkaline suspension underwent centrifugation at 3500 rpm. After supernatant removal, the chitosan pellets were washed repeatedly with distilled water until a neutral pH was reached. To eliminate potential traces of lipids, proteins, or pigments, the chitosan pellets were washed with a 1:1 mixture of ethyl alcohol and acetone, using a ratio of 1:10 (*m*/*v*) between the wet chitosan pellets mass and the solvent mixture volume. The organic solvents were then removed through centrifugation and multiple washings with distilled water. Finally, the resulting wet chitosan pellets were dried in an oven at 105 °C. All the chitosan powders obtained were stored in a desiccator at room temperature, readying them for physicochemical characterization and the preparation of test solutions.

MM and DDA of polymer molecules are the essential features that influence most structure–function relationships. Therefore, the DDA characterization was carried out using potentiometric pH measurements [30,74], in triplicate. Equations (3) and (4) were used for the determination of DDA values:

(3) $$\mathrm{DDA}\;\left(\%\right)=\frac{203\cdot Q}{1+42\cdot Q}$$

(4) $$Q=\frac{C_{M}\cdot \Delta V}{m}$$

where C_M_ is the molar concentration of the NaOH solution used for titration (mol/L); ΔV represents the difference volume between the two inflexion points (L); m is the mass of chitosan analyzed (g); 203 is a coefficient that represents the molar mass of chitin (g/mol); and 42 is a coefficient that represents the molar mass of acetyl group (g/mol).

The molar mass (MM) was determined by measuring the intrinsic viscosity of diluted acid solutions of chitosan prepared in 2% acetic acid with 0.1 M KCl [86] using an Ostwald-type capillary viscometer (model 518 10, SI Analytics GmbHTM, Mainz, Germany, capillary tube inner diameter Øi = 0.43 mm) [30,83]. The measurements were carried out at 25 ± 1 °C, in triplicate. The approach employed in this study relies on the correlation between the viscosimetric-average molar mass, MM (g/mol), and the intrinsic viscosity, [η] (mL/g), as described by Mark–Houwink–Sakurada [30,83] and illustrated by Equation (5).

(5) $$\left[\eta \right]=K\cdot M_{v}^{\alpha }$$

In this equation, the constants K and α are influenced by factors such as the solvent’s characteristics, temperature, and the chemical arrangement of the polymer (K = 13.8 × 10^−3^ mL/g, α = 0.85) [52,86].

For testing, chitosan powder samples (CHP1, CHP2, CHP3) were mixed with oligochitosan powder in mass ratios of 1:2 (m/m) and then solubilized in dilute 1% acetic acid solutions. The powders of α-chitosan, β-oligochitosan, and their mixtures were solubilized on a hotplate under continuous medium stirring (500 rpm) at 40 °C for 24 h.

The characterization was performed using a scanning electronic microscope (SEM) (FEI QUANTA 250) with an energy-dispersive spectrometer. Images with a magnitude of 500× and 2000× and details of the microparticle disposition were photomicrographed.

### 4.3. Antioxidant Activity

The antioxidant activity of the samples was determined by the DPPH (2,2-diphenyl-1-picryl-hydrazyl-hydrate) method. DPPH (Sigma Aldrich) in methanol solutions (100 mL) were prepared before performing the test (fresh solution). Fresh chitosan solutions in 1% acetic acid were prepared. The samples were stirred for two hours before assay and after that, they were kept warm at 37 °C. A volume of 100 µL of the sample (35 µg/mL concentration), including chitosan solutions (CHP1, CHP2, CHP3) and mixtures (MIX1, MIX2, MIX3), were mixed with 1 mL of DPPH. The solutions were stirred for 10 s and kept at the thermostat (37 °C) in the dark for 30 min. The evaluation was carried out with a UV/VIS Nanospectrophotometer, NABI (MicroDigital Co., Ltd., Seongnam-si, Republic of Korea), and the reading was carried out at 517 nm. The DPPH solution was used as control, and methanol was used as a blank. The free radical’s scavenger activity of chitosan solutions was calculated as follows (Equation (6)):

(6) $$\mathrm{DPPH}\;\left(\%\right)=\frac{A_{\mathrm{control}}-A_{\mathrm{sample}}}{A_{\mathrm{control}}}\cdot 100$$

where A_control_ and A_sample_ represent the absorbance values at 517 nm [61].

All variants were completed in triplicate. The results are presented as an average value with standard deviation (±SD).

### 4.4. Cell Culturing Conditions

Eagle’s Minimum Essential Medium (Eagle’s Minimum Essential Medium, PAN-Biotech GmbH, Aidenbach, Germany) was used for the SK-MEL-28 cell line, with both types of nutrient media supplemented with 10% fetal bovine serum (Euroclone S.p.A., Milan, Italy) and 1% antibiotic solution (mixture of penicillin 100 μg/mL and streptomycin 100 IU/mL—Capricorn Scientific GmbH, Ebsdorfer-grund, Germany). Cells were grown in culture plates in an incubator (Binder GmbH, Tuttlingen, Germany) at 37 °C in a humid atmosphere with 5% CO_2_ [87].

SK-MEL-28 cells were cultured in the presence of chitosan solutions (CHP1, CHP2, CHP3) of different concentrations (4.375, 8.75, 17.5, and 35 µg/mL) and mixtures of chitosan–oligochitosan MIX1 (CHP1 + COP4), MIX2 (CHP2 + COP4), and MIX3 (CHP3 + COP4).

### 4.5. MTT Assay

The MTT assay, which allows the evaluation of the percentage of cell viability, was used in order to determine optimal doses to express the biological effect of the tested compounds, based on which the IC_50_ value was calculated. The 3-(4,5-dimethylthiazol-2-yl)-2,5-diphenyl tetrazolium bromide (MTT) colorimetric method was used, adapted from Mosmann [88], Laville et al. [89], and van Meerloo et al. [90]. This is based on the ability of living cells to convert the yellow water-soluble substrate of MTT, with the help of mitochondrial dehydrogenases, into dark, blue-colored formazan. Briefly, 10 µL of DMSO in each well was added in order to solubilize formazan crystals and to stop the reaction. The amount of formazan produced is hypothesized to be directly proportional to the number of living cells [91].

Initially, SK-MEL-28 cells were grown in 75 cm^2^ culture flasks and, after monolayer formation, were detached with trypsin/EDTA, counted, and resuspended in 96-well microplates at a density of 1 × 10^4^ cells/well. After forming the monolayer (approximately 24 h), the cells were treated with the formulas of α-chitosan and β-oligochitosan in the following doses: 4.375, 8.75, 17.5, and 35 µg/mL, with the duration of treatment being 24 and 48 h. A 1% acetic acid solution was used to solubilize the powdered chitosan.

At the end of the treatment, the cells were processed following the protocol of the MTT test, and the extinctions were measured at 570 nm using the automatic microplate reader Biochrom EZ Read 400 (Biochrom Ltd., Cambridge, UK). Cell viability was calculated according to Equation (7).

(7) $$\mathrm{Cell}\;\mathrm{viability}\;\left(\%\right)=\left(E_{\mathrm{sample}}/E_{\mathrm{control}}\right)\cdot 100$$

where E_sample_ is the extinction of the sample and E_control_ is the extinction of the untreated control.

The IC_50_ values of the tested chitosan formulations were calculated using polynomial dose–response curve plots for each concentration used in the MTT assay.

### 4.6. Cell Morphology Test

The interaction of different chitosan formulas with cellular structures was also evaluated regarding the morphological changes they induced after 24 and 48 h of treatment. Cell morphology was visualized using a Nikon Eclipse TS100 inverted microscope equipped with a MshOt MS60 (Nikon, Tokyo, Japan) digital camera, with photographs taken with a 10× objective.

### 4.7. Determination of YKL-40 Protein Expression

The melanoma cell line SK-MEL-28 was used to evaluate the expression of the chitinase-like YKL-40 protein. Eagle growth medium (Eagle’s Minimum Essential Medium) was supplemented with 10% fetal bovine serum and 1% antibiotic solution. Cells were grown in culture flasks in an incubator (Binder GmbH, Tuttlingen, Germany) at 37 °C in a humid atmosphere with 5% CO_2_ [87].

The evaluation of the expression levels of chitinase-like proteins, especially YKL-40, was performed by the ELISA test. The test was initiated by culturing SK-MEL-28 cells in 150 cm^2^ plates to obtain cellular mass, after which the cells were trypsinized with trypsin/EDTA, counted, and distributed in 12-well plates at a density of 3.6 × 10^4^ cells/well. After the formation of the monolayer (24 h), the growth medium was removed and replaced with fresh medium; the cells were stimulated with 50 ng/mL of lipopolysaccharide (LPS, MedChemExpress, Monmouth Junction, NJ, USA) for one hour, after which they were treated for 24 h with chitosan and chitosan–oligochitosan mixtures. The concentrations used are for each compound tested, concentrations that induce similar effects (IC_50_). After the treatment ended, the supernatant was collected and processed according to the specific instructions of the ELISA kit. To calculate the results expressed in pg/mL, a standard curve consisting of 8 samples with known protein concentration was drawn up. The results were expressed in percentages (%), and the alterations in the chitosan-exposed samples (mean values) were compared with the control sample (LPS stimulated control).

### 4.8. Statistical Analysis

Results of in vitro tests were expressed as mean ± standard error (SE). The difference between mean values for each index was expressed using Student’s *t*-test [92]. Statistical significance was established at *p* < 0.05; each test was performed in triplicate. All the registered data were analyzed using multi-ANOVA implemented in Matlab 2023, checking the influence of the three factors implied: duration of the treatment; concentration; and polymer type.

## 5. Conclusions

The results obtained provide promising information on modeling the efficiency of antitumor action. Thus, α-chitosan, with different degrees of deacetylation (DDA) and molar mass (MM), in combination with β-oligochitosan, can form mixtures with favorable effects on the cell processes, activating antitumor mechanisms by disrupting cellular activity and maintaining the nonproliferative status. Our study showed decreased tumor cell viability and YKL-40 expression in the association conditions of α-chitosan–β-oligochitosan mixtures after cell stimulation. These results highlight the importance of obtaining carefully controlled solutions from a molecular point of view that correlate with the targeted cellular processes. We appreciate that DDA is a critical factor in relation to the extent of the effects, while MM influences the conditions of the chitosan penetration and, implicitly, the rate of the induced reactions.

The results support the conclusion that the chitosan mixtures determined a dose- and duration-dependent cellular reactivity. The intensity of the response was correlated with the degree of damage to cell viability. The morphological changes highlighted the decrease in the ability to adhere and, implicitly, to form the cell monolayer.

Our future studies aim to investigate the mechanisms by which these mixtures of another α-chitosan–β-oligochitosan combination ratio can better amplify the antiproliferative effect in melanocytes and other tumoral cell lines.

## Figures and Tables

**Table 1:** Physicochemical characteristics of tested samples.

Sample Code	DDA (%)	MM (kDa)	Chitosan Conformation
CHP1	96.27	819.99	α-chitosan
CHP2	86.65	804.33	α-chitosan
CHP3	88.50	475.43	α-chitosan
COP4	70.00	1.50	β-oligochitosan
MIX1(mixture CHP1 + COP4) *	78.76	2.25	α-chitosan and β-oligochitosan (1:2)
MIX2 (mixture CHP2 + COP4) *	75.55	2.25	
MIX3 (mixture CHP3 + COP4) *	76.17	2.25	

* Calculated with Equations (1) and (2), respectively.

**Table 2:** Evaluation of antioxidant activity (%) for samples used in SK-MEL-28 cell culture assay (CHP1, CHP2, CHP3, COP4, MIX1, MIX2, MIX3); DPPH assays were evaluated at 37 °C.

Samples	Antioxidant Activity (%), ± SD
CHP1	13.28 ± 1.59
CHP2	18.79 ± 1.04
CHP3	15.15 ± 0.05
COP4	11.57 ± 0.11
MIX1	14.27 ± 1.81
MIX2	10.52 ± 0.16
MIX3	11.63 ± 1.37

**Table 3:** Inhibition concentrations (IC_50_) values after treatment application with various formulas of chitosan evaluated.

Samples	IC_50_ 24 h (µg/mL)	IC_50_ 48 h (µg/mL)
CHP1	88.74	62.91
CHP2	78.45	47.01
CHP3	73.98	64.58
MIX1	75.01	59.21
MIX2	73.40	53.06
MIX3	75.01	64.22

**Table 4:** Analysis of variance in three-way ANOVA.

Source of Variation	SS	df	MS	F	*p*-Value
Time	685.58	1	685.58	51.58	0
Concentration	2927.71	3	975.90	73.43	0
Polymer	238.21	5	47.64	3.58	0.0248
Time × Concentration	28.18	3	9.39	0.71	0.5628
Time × Polymer	305.56	5	61.11	4.60	0.0096
Concentration × Polymer	299.70	15	19.98	1.50	0.2195
Within	199.37	15	13.29		
Total	4684.31	47			

**Table 5:** Analysis of variance for samples at 24 h contact time.

Source of Variation	SS	df	MS	F	*p*-Value
Polymers	284.46	5	56.89	3.69	0.0066
Concentration	3065.01	3	1021.67	66.31	0.0000
Interaction	389.28	15	25.95	1.68	0.0867
Within	739.55	48	15.41		
Total	4478.30	71			

**Table 6:** ANOVA results for melanoma cells’ viability using the investigated biopolymers for a contact time of 48 h and solution concentration 4.375 µg/mL (a), solution concentration 8.75 µg/mL (b), solution concentration 17.5 µg/mL (c), and solution concentration 35 µg/mL (d).

**(a)**	**Source of Variation**	**SS**	**df**	**MS**	**F**	***p*** **-Value**
	Between Groups	936.24	5	187.2	7.91	0.00167
	Within Groups	284.03	12	23.67		
	Total	1220.26	17			
(**b**)	**Source of Variation**	**SS**	**df**	**MS**	**F**	***p*** **-Value**
	Between Groups	616.52	5	123.3	6.52	0.00375
	Within Groups	226.89	12	18.91		
	Total	843.41	17			
(**c**)	**Source of Variation**	**SS**	**df**	**MS**	**F**	***p*** **-Value**
	Between Groups	198.82	5	39.76	2.15	0.12908
	Within Groups	222.20	12	18.52		
	Total	421.03	17			
(**d**)	**Source of Variation**	**SS**	**df**	**MS**	**F**	***p*** **-Value**
	Between Groups	226.83	5	45.37	2.17	0.12598
	Within Groups	250.80	12	20.9		
	Total	477.63	17			

## Data Availability

Data is contained within the article.
